# Spatial2GWAS: a database for linking spatial transcriptomic regions with GWAS traits

**DOI:** 10.1093/nar/gkaf1047

**Published:** 2025-10-22

**Authors:** Xi Hu, Aoqi Wang, Huan Yu, Pora Kim, Xiaobo Zhou

**Affiliations:** West China Biomedical Big Data Center, West China Hospital, Sichuan University, Chengdu, Sichuan 610041, P.R. China; West China Biomedical Big Data Center, West China Hospital, Sichuan University, Chengdu, Sichuan 610041, P.R. China; West China Biomedical Big Data Center, West China Hospital, Sichuan University, Chengdu, Sichuan 610041, P.R. China; Center for Computational Systems Medicine, McWilliams School of Biomedical Informatics, The University of Texas Health Science Center at Houston, Houston, TX 77030, USA; Center for Computational Systems Medicine, McWilliams School of Biomedical Informatics, The University of Texas Health Science Center at Houston, Houston, TX 77030, USA

## Abstract

Spatial heterogeneity of gene expression within tissue regions has a critical influence on biological functions, thereby affecting disease pathogenesis. However, systematic associations between spatially resolved transcriptomes and phenotypes, especially in complex diseases, remain underexplored. Here, we developed spatial2GWAS (http://www.spatial2gwas.cn), a comprehensive resource linking spatial transcriptomic (ST) regions with GWAS traits. In the database, we collected 1196 ST slices (human and mouse) from five technologies and 812 GWAS traits spanning 18 phenotype categories and identified 29 701 ST slice-GWAS trait pairs containing 47 492 significant regions. Functional analyses reveal distinct patterns of cell type composition, gene expression, GO/KEGG pathway activation, and cell–cell communication direction between trait-related and unrelated spatial regions. The database provides a user-friendly interface for visualization of spatial regions and GWAS trait associations, supporting advanced queries by slice and GWAS information, genes co-expressed with GWAS trait-associated genes, and spatial regions. Spatial2GWAS aims to enable systematic exploration of spatial mechanisms underlying complex traits and offer insights into region-specific biological functions and potential therapeutic targets. This database bridges ST and high-level phenotypes, advancing the understanding of tissue heterogeneity in complex human diseases.

## Graphical Abstract

**Graphical Abstract:**
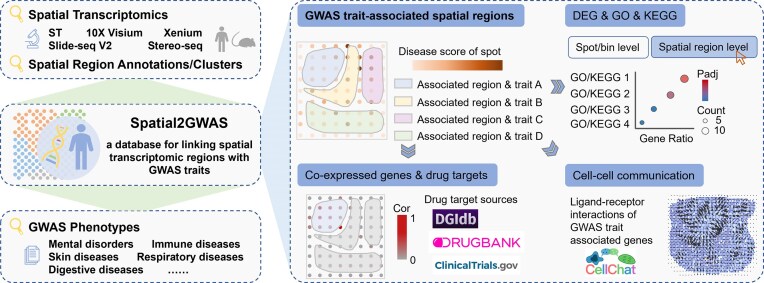

## Introduction

Spatial transcriptomics (ST) has revolutionized the understanding of tissue architecture by investigating region-specific gene expression patterns, offering critical insights into cell localization, microenvironment interactions, and spatial heterogeneity in biological processes [1]. These features are particularly vital in elucidating the molecular mechanisms underlying complex human diseases such as cancer, neurological disorders, and developmental pathologies [2, 3]. Genome-wide association studies (GWAS) have identified various loci linked to complex traits by leveraging large-scale population cohorts. However, the functional interpretation of trait-associated single nucleotide polymorphisms (SNPs) remains challenging, as a significant proportion of variants are located in noncoding regions, and individual variants often exhibit weak effects [4]. The joint association of SNPs at the gene level has been suggested as a more powerful alternative to inferring gene effects on phenotypes based on single SNP statistics in GWAS, and several algorithms have been widely used to identify GWAS trait-associated genes [5–7]. In a recent study, Song *et al.* developed a new approach to estimate spatial genotyping from ST data of the brain and embryo, advancing the integration of genomics and spatial information [8]. A further integration of GWAS trait-associated genes with ST data enables the dissection of spatially resolved region distribution related to high-level phenotypes, enhancing the discovery of pathological, prognostic, and therapeutic targets by anchoring gene associations to specific tissue contexts [9].

Recent studies have highlighted the importance of spatially differential gene expression in diseases [10–12]. For example, glutamatergic neurons distributed near the dorsal hippocampus were found to be associated with schizophrenia, while those near the deep medial prefrontal cortex were associated with depression [8]. Miyoshi *et al.* identified cortical layer L3/L4 related to Alzheimer’s disease (AD) through human and mouse ST data, coinciding with L3-preferential amyloid deposition [13]. These findings demonstrate that combining spatial context can enhance the power to detect trait-associated tissue regions and cell types. However, there is still a lack of a comprehensive database focused on investigating trait-associated spatial regions by integrating ST with GWAS data. Existing ST databases, such as SPASCER [14], SpatialRef [15], stSNV [16], and SPathDB [17], primarily carry spatial annotation and biological functional characterization but lack a direct link between spatial features and high-level phenotypes. Sc2GWAS [18] prioritizes cell-type-level GWAS associations but ignores the spatial localization of cells, which is critical for realizing cell functions in specific tissue contexts. These limitations underscore the urgent need for a comprehensive resource that systematically integrates ST and GWAS data to reveal the spatial heterogeneity across phenotypes.

To address these gaps, we developed spatial2GWAS, a database that systematically links ST with GWAS traits across tissues accessible at http://www.spatial2gwas.cn. By matching 1196 ST slices from human and mouse samples derived from five technologies and 812 GWAS traits of 18 phenotype categories, we identified 47 492 spatial regions significantly associated with GWAS traits. The database provides a user-friendly web interface for exploring and visualizing differences between GWAS trait-related and unrelated spots/bins and spatial regions, including cell-type composition, gene expression, GO/KEGG term enrichment, and directional cell–cell communication. The spatial2GWAS database not only enhances the discovery of region-specific biological features and actionable targets for diseases by integrating spatial resolution gene expression and population-scale genetics, but also provides a platform for hypothesis generation in precision medicine and disease modeling.

## Materials and methods

### Collection and processing of ST data

For ST data involved in spatial2GWAS, we collected ST slices of human and mouse samples from SPASCER [14], SpatialRef [15], stSNV [16], and STOmicsDB [19] databases (Supplementary Tables S1 and S2). These slices were sequenced on ST platforms of low-resolution traditional ST and 10× Visium, as well as high-resolution Stereo-seq, Slide-seq V2, and Xenium. The spots in ST data from low-resolution technologies and bins from high-resolution technologies were taken as basic elements in this study. Annotations of spatial region types of spots/bins for 986 ST slices were collected from the SpatialRef database and original publications. For the other 210 ST slices across 23 tissues sequenced by 10× Visium from the stSNV database that do not have region annotations, we used the Building Aggregates with a Neighborhood Kernel and Spatial Yardstick (BANKSY) method (version: 1.2.0) [20] to cluster spots/bins under the suggested parameters of “lambda = 0.2” and “resolution = 0.5,” based on gene expression and spatial distance features to segment tissue regions. The cell type compositions by deconvolution analysis were downloaded to compare biological differences across clusters. For each ST slice, three matrices of gene expression, spatial coordinates, and spatial annotations were matched by barcode IDs of spots/bins and saved in h5ad format. Only spots/bins with gene counts >250 and genes that were expressed in >50 spots/bins were kept for further analyses. The pre-processed gene expression count matrix was normalized and transformed into log values using Scanpy (version: 1.10.4) [21] for downstream analysis. After quality control, the spatial2GWAS database contains 1196 ST slices (404 from humans and 792 from mice) across 39 tissues, such as spinal cord, brain, skin, and kidney, and encompasses both healthy and diseased states.

### Collection and processing of GWAS summary data

The public GWAS summary data were collected from the sc2GWAS [18] and DMRdb [22] databases, which were from a range of public datasets such as Neale Lab UKBB v3 (https://www.nealelab.is/uk-biobank), GWAS Catalog [23], and FinnGen [24]. Strict quality control was carried out to filter confident datasets in the original studies. In total, we obtained 812 human GWAS summary data across 18 phenotype categories, such as mental disorders, immune diseases, and skin diseases (Supplementary Tables S3 and S4). For each GWAS summary data, the columns of dbSNP ID (rsID), chromosome, position, sample number, beta, and *P*-value were extracted. SNP positions were converted to GRCh38 using LiftOver [25] if they were mapped to other versions of the human genome references. The linkage disequilibrium between SNPs was downloaded from the 1000 Genomes project of European, East Asian, and African populations. Based on the positions of SNPs and genes and SNP associations with GWAS traits from the GWAS summary data, the Multi-marker Analysis of GenoMic Annotation (MAGMA) method (version: 1.10) [5] was used to estimate gene-level associations with phenotypes. In detail, MAGMA first mapped SNPs tested in at least 50 samples within 100 kb surrounding regions onto genes and then calculated *z*-scores of genes and ranked them to form the list of top 1000 GWAS trait-associated genes for each GWAS summary dataset.

### Identification of associations between spatial regions and phenotypes

To identify spatial regions associated with GWAS traits, ST data were first matched with GWAS summary data by tissue type. For example, ST slices from the brain were paired with GWAS traits of mental disorders and neurological diseases. This ensures that analyses are biologically meaningful by linking relevant tissue-specific gene expression with trait-associated genetic markers. For each ST slice-GWAS trait pair, GWAS summary data and ST data were connected through genes. In the GWAS trait data, MAGMA assigned genetic markers to genes to obtain the top 1000 genes associated with the phenotype. The single-cell disease relevance score (scDRS) approach (version: 1.0.3) [26] was employed to quantify the expression levels of these GWAS trait-associated genes on the paired ST slice and analyze associations between spots/bins and the GWAS trait. In detail, scDRS first calculated a disease score for each spot/bin based on the MAGMA *z*-score weighted expression of the top 1000 GWAS trait-associated genes. Then, control scores were generated by randomly selecting 1000 genes 1000 times to establish a background distribution. The distribution of the disease score and control scores was used to calculate a *P*-value for each spot/bin, indicating the enrichment of GWAS trait-associated genes relative to the background gene expression. Spots/bins with *P*-values < .05 were considered to be associated with the GWAS trait in this study. These spot/bin-GWAS trait associations were then aggregated to the spatial region level. The distribution of the top 5% quantile of disease and control scores of spots/bins within each region was used to calculate a *P*-value to evaluate the association between the spatial region and the trait. The *P*-values of spatial regions on the ST slice were adjusted using the Benjamini & Hochberg method, and spatial regions with adjusted *P*-values < .05 were considered as GWAS trait-related regions. For each ST slice-GWAS trait pair, the results include *P*-values, adjusted *P*-values, and *z*-scores for spatial regions, which were used to assess statistical significance and prioritize associations. The associations were visualized using spot/bin and region coordinates, providing a spatial representation of the relationship between tissue regions in ST data and the GWAS trait.

### Identification of co-expressed genes and drug targets

The disease scores calculated by scDRS represent a combined expression level of GWAS trait-associated genes. To obtain potential genes co-expressed with GWAS trait-associated genes, we performed Pearson’s correlation analysis of gene expression and the normalized disease scores in trait-related spots/bins in each pair of ST slice and GWAS trait using R software (version: 4.4.1). Co-expressed genes were identified under a threshold of Benjamini & Hochberg adjusted *P*-values < .05. The analysis prioritizes disease-relevant genes by evaluating transcriptomic gene expression and genomic disease score. To investigate potential drug targets for diseases with spatial characteristics, we overlapped the co-expressed genes with drug targets from DGIdb (version: 5.0) [27]. We also collected the experimental and investigational information from the DrugBank database (version: 5.1.13) [28] and the clinical trials information from the ClinicalTrials.gov database (https://clinicaltrials.gov/, collected by 30 June 2025) to provide a clear overview of the application of the corresponding drugs in clinical settings.

### Identification of differentially activated biological functions

To investigate differentially activated biological functions for GWAS trait-associated spatial distributions, we identified differentially expressed genes (DEGs) between GWAS trait-related and unrelated spots/bins and trait-related and unrelated spatial regions using Seurat (version: 5.2.1) [29]. Genes were considered to be significant with an absolute log2FC (Fold Change) > 0.25 and Benjamini & Hochberg adjusted *P*-values < .05 through the Wilcoxon test between the related and unrelated groups. Based on DEGs, Gene Ontology (GO) terms (including biological process, molecular function, and cellular component) [30, 31] and Kyoto Encyclopedia of Genes and Genomes (KEGG) pathways [32] were enriched using the clusterProfiler (version: 4.14.6) [33] with a significant threshold of Benjamini & Hochberg adjusted *P*-values < .05. The top 15 enriched GO terms and KEGG pathways were visualized using bubble charts to show adjusted *P*-values and ratios of DEGs in the GO/KEGG gene sets.

### Cell–cell communication signaling of GWAS trait-associated genes

Cell–cell communication is mainly mediated by biochemical signaling of ligand-receptor binding to shape development, structure, and function in the downstream. The distance features between cells or bins in ST data help to infer cell–cell communication that takes place within limited spatial distances, instead of only considering a few cell types. In the spatial2GWAS database, we used the COMMunication analysis by Optimal Transport (COMMOT) method [34], which accounts for ligand-receptor competition and spatial distances, to construct directional cell–cell communication signals and investigate signaling within and between GWAS trait-related and unrelated spatial regions. The ligand-receptor pairs in human and mouse were collected from the CellChat database [35]. The ligand-receptor pairs were kept for analysis if the ligand or receptor belonged to GWAS trait-associated genes and their co-expressed genes, and the ligand and receptor were detected in >5% of spots/bins. The cell–cell communication results were visualized using scatter plots, with nodes representing spots/bins on ST slices, node color representing sender weight, and arrows showing signaling directions.

### Database architecture

The spatial2GWAS database is freely available at http://www.spatial2gwas.cn/. The system is constructed based on a three-tier architecture: client, server, and database. The front end of spatial2GWAS was developed using Vue 3 (https://vuejs.org/). The back end of spatial2GWAS was developed using Node.js (version: 22, https://nodejs.org/zh-cn/) and Django (version: 5.0.1, https://www.djangoproject.com/). Data management was performed using MySQL (https://www.mysql.com/). Interactive graphs for data statistics were created by ECharts (version: 4.0, https://www.echartsjs.com/). The user-friendly interface of spatial2GWAS is compatible with popular web browsers, such as Google Chrome, Firefox, and Microsoft Edge, and is accessible and readable on phone and tablet screens. All results of spatial2GWAS were uploaded to Zenodo and can be downloaded at https://zenodo.org/records/16880800.

## Results

### Overview of spatial2GWAS

**Figure 1.**
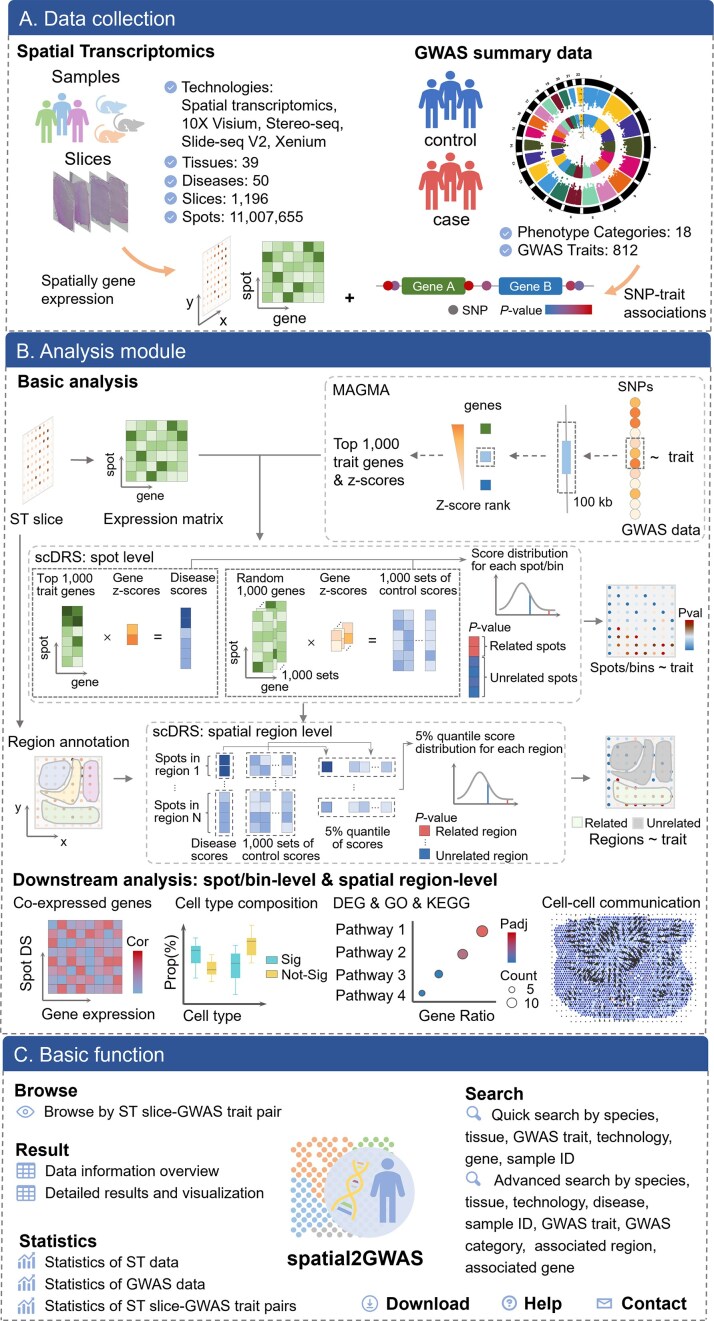
Overview of spatial2GWAS. (**A**) Data resources and overview in spatial2GWAS of ST slices and GWAS summary statistics. (**B**) Analysis modules in spatial2GWAS, including ST and GWAS data integration, the basic analysis of the scDRS pipeline, and downstream analysis at both the spot/bin level and spatial region level. (**C**) Basic function of spatial2GWAS. Spatial2GWAS mainly provides browse, search, download, and statistics functions. DS: disease score.

The spatial2GWAS provides associations between spatial regions and GWAS traits and results of downstream analysis for biological characteristics, as outlined in the pipeline shown in Fig. 1. In total, the database contains 1196 ST slices with 11 007 655 spots/bins across 39 tissues. The ST data were matched with 812 GWAS traits by tissue type to compose candidate ST slice-GWAS trait pairs (Fig. 1A). Using the scDRS pipeline, we identified significant associations at both spot/bin and spatial region levels for ST slices and GWAS traits. To investigate the underlying mechanisms of spatial distribution within tissues toward high-level phenotypes, especially complex diseases, we carried out downstream analysis at spot/bin and spatial region levels, including the identification of co-expressed genes with GWAS trait-associated genes, annotation of drug targets for co-expressed genes, comparison of cell type composition, enrichment of GO/KEGG processes based on DEGs, and inference of spatial direction of cell-cell communication (Fig. 1B). All data and analysis results are archived in the spatial2GWAS database, enabling users to explore spatial features related to high-level phenotypes through data browsing, searching, and visualization (Fig. 1C). The spatial2GWAS database is a valuable resource for enhancing the biological interpretability of GWAS results in the spatial context and highlighting potential spatially specific effects in tissues on complex diseases.

### Browsing ST spatial region and GWAS trait associations

#### Browse interface

**Figure 2.**
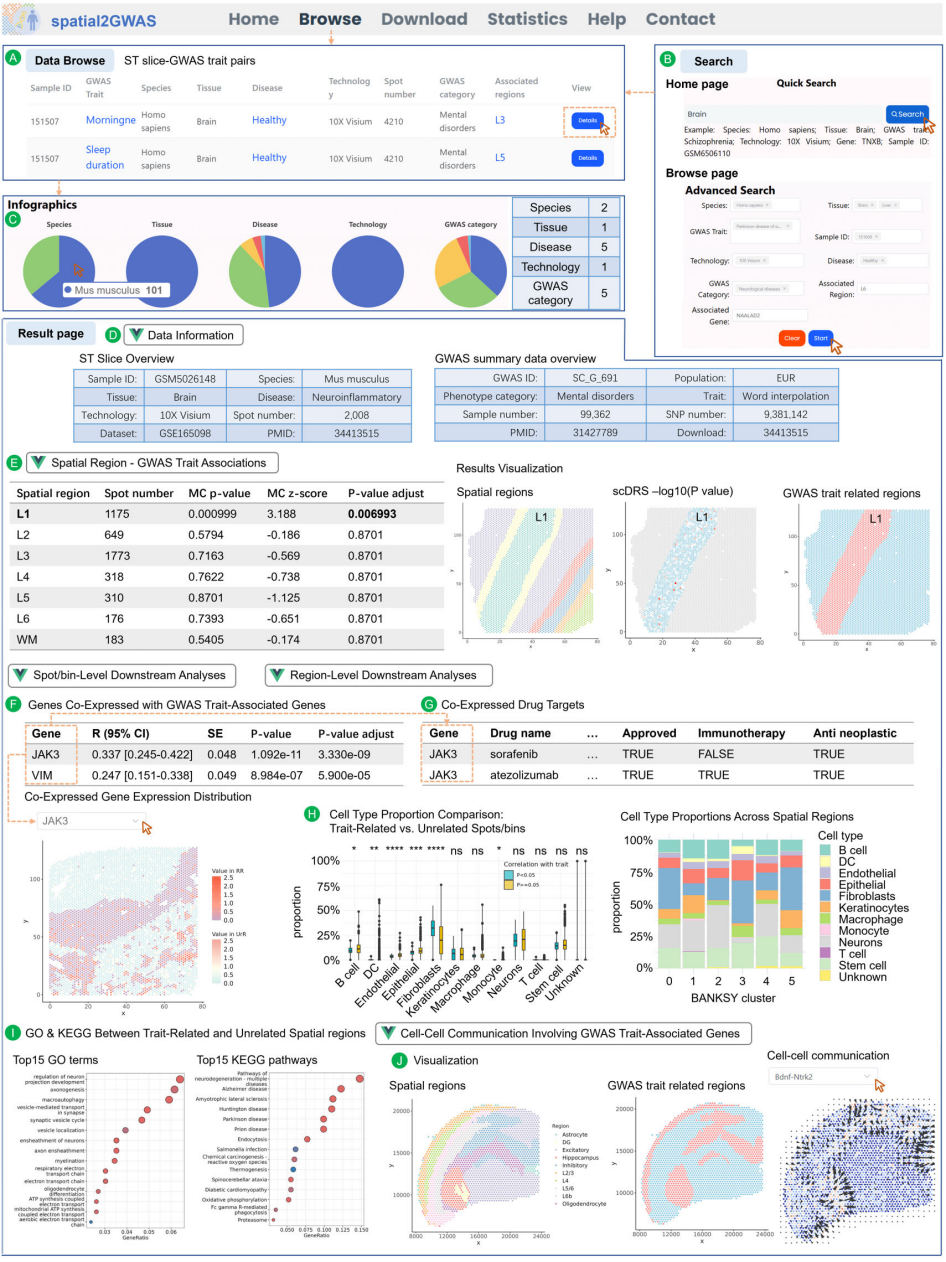
Functions and usage of spatial2GWAS. (**A**) Overview of ST slice-GWAS trait pairs. Each row includes metadata for the ST slice (“Sample ID,” “Species,” “Tissue,” “Disease,” “Technology,” and “Spot number”), GWAS summary data (“GWAS trait” and “GWAS category”), and “Associated regions” result in the pair. (**B**) Quick search on the “Home” page and advanced search on the “Browse” page. (**C**) Infographics and summaries displayed after filtering by “Tissue” (“Brain”) and “Technology” (“10× Visium”). (**D**) Data overview of the selected ST slice and GWAS summary data. (**E**) Associations between human brain spatial region L1 and GWAS trait “AD.” Visualization includes: spatial region annotations (left), *P*-values of spots in the trait-related region calculated by scDRS (middle), and spatial region related and unrelated to the GWAS trait (right) (sample ID: 151510). (**F**) Expression distribution of the co-expressed gene JAK3 in a liver cancer ST slice (Sample ID: HCC-3L) and GWAS trait “Cancer (diagnosed by doctor)” pair. RR: GWAS trait-related regions; UrR: GWAS trait-unrelated regions. (**G**) Examples of drug targets identified among co-expressed genes in a liver cancer ST slice (Sample ID: HCC-3L) and GWAS trait “Cancer (diagnosed by doctor)” pair. (**H**) Visualization of cell type proportion differences between GWAS trait-related and unrelated spots (left, box plot) and cell type proportions in spatial clusters (right, stacked bar plot) (Sample ID: GSM4565823; GWAS trait: “Diagnoses—main ICD10: C44 Other and unspecified malignant neoplasm of skin”). (**I**) Bubble charts showing the top 15 enriched GO terms and KEGG pathways based on DEGs between GWAS trait-related and unrelated spatial regions (Sample ID: 151673; GWAS trait: “AD”). (**J**) Spatial region annotations in a mouse brain ST slice (left), trait-related regions (middle), and directional cell–cell communication results of the Bdnf-Ntrk2 interaction generated by COMMOT (right) (Sample ID: 37864231_brain_02; GWAS trait: “chronotype”).

The spatial2GWAS database provides interfaces for browsing and searching all ST spatial region and GWAS trait associations, along with visualization of corresponding downstream analysis results. On the “Browse” page, a table lists 29 701 ST slice-GWAS trait pairs with significant spatial regions related to GWAS traits (12 668 pairs from human and 17 033 pairs from mouse), and each row includes basic information of the ST slice, GWAS data, and associated regions in the pair (Fig. 2A). Users can filter the table using category options for ST and GWAS data as keywords in the “Advanced Search” box, as well as “Associated Region” and “Associated Gene” (symbols of genes co-expressed with GWAS trait-associated genes). Multiple filters can be applied simultaneously using the “Start” button and cleared at any time using the “Clear” button. Additionally, the “Quick Search” box and labels on the “Home” page also enable filtering and direct users to the filtered table of ST slice-GWAS trait pairs (Fig. 2B). The “Infographics” section dynamically and interactively displays statistical summaries of the ST slice-GWAS trait pair table, both before and after filtering on the “browse” page. These summaries include partial and total counts for “Species,” “Tissue,” “Disease,” “Technology,” and “GWAS category,” helping users gain an overview of the results (Fig. 2C). When users click the “Detail” button in the “View” column of the table, the detailed analysis results for the corresponding ST slice-GWAS trait pair are displayed on a new page.

#### Sample overview

On the result page for the selected ST slice-GWAS trait pair, tables in a drop-down menu labeled “Data Information” first provide detailed descriptions of the ST slice and the GWAS summary data (Fig. 2D). Users can access complementary information of the dataset and publication for the ST slice, as well as the population, sample size, total number of SNPs, download link, and publication for the GWAS data, to retrieve the original data conveniently.

#### Associations between spatial regions and GWAS trait

We used the scDRS pipeline by evaluating the association between each spot/bin and the trait and integrating spots/bins based on region annotations to derive the associations between spatial regions and the trait. In the current version of spatial2GWAS, we identified a total of 47 492 significant GWAS trait-related spatial regions. For the selected ST slice-GWAS trait pair, the result page provides a “Spatial Region-GWAS Trait Associations” section, presenting a summary table of associations between spatial regions and the GWAS trait, including spatial region names, spot/bin counts of each region, and adjusted *P*-values. In this section, we also provide three plots to visualize the location of annotated spatial regions on the ST slice, *P*-values of GWAS trait association for spots/bins involved in the analysis, and classifications of trait-related and unrelated spatial regions. Users can quickly acquire the spatial distribution of regions related to the GWAS trait in the tissue and further build hypotheses of interactions within and across spatial regions based on knowledge about the trait.

For example, in four ST slices from healthy human brain samples, we identified an association between the cortical Layer 1 region and the GWAS trait of AD (Supplementary Fig. S1 and Fig. 2E). Layer 1 is the cortex’s uppermost layer and densely packed with neurons, such as excitatory neurons, long-range projection neurons, and cortical inhibitory interneurons, to convey sensory and behavioral information [36, 37]. A study found that the activity of inhibitory neurons in Layer 1 during early-stage AD drives hyperactivation of Layer 2/3 excitatory neurons, enhancing Aβ production and forming a pathological cascade linked to cognitive decline [38]. The similarity in expression patterns between genes in Layer 1 in the healthy brain and AD-associated genes may indicate a greater susceptibility to early AD in Layer 1 compared to deeper cortical layers. These examples indicate that spatial2GWAS could provide reliable resources to investigate spatial features of tissues in complex diseases.

#### Co-expressed genes and drug targets

Genes co-expressed with GWAS trait-associated genes in trait-associated spots/bins help to investigate spatial patterns of gene expression contributing to the high-level phenotype. Across 17 577 ST slice-GWAS trait pairs in the spatial2GWAS database, we identified 15 022 human genes and their homologs in mice co-expressed with GWAS trait-associated genes. The results are presented in the “co-expressed gene expression distributions” subsection on the result page. Spatial expression pattern visualizations of these co-expressed genes are displayed in the “co-expressed gene expression distributions” subsection, selected by gene symbol. Additionally, we identified candidate drugs by overlapping co-expressed genes with known drug targets in clinical treatment and experiments. These genes were mapped to 3216 drug targets linked to 11 986 drugs. Interestingly, among these, 3242 are FDA-approved drugs, 2522 are investigational drugs, 332 are experimental drugs, and 3622 have been tested in clinical trials. The drug target mappings are detailed in the “co-expressed drug targets” subsection. Users can compare potential differences of drug sensitivity across spatial regions, especially drugs that may contribute to the treatment of the region-associated complex diseases. The results may give a clue to choose specific drugs or combination therapy based on tissue region characteristics.

In an ST slice from the leading-edge section of a liver cancer patient, spatial regions annotated as “immune” and “stromal” were associated with the GWAS trait “Cancer (diagnosed by doctor)” (Supplementary Fig. S2). Cancer-related genes such as JAK3 and VIM were upregulated with the increased level of trait-associated genes in spots [39, 40]. Correlation analysis results and gene expression distributions are displayed in the “Spot/bin-level downstream analyses” section (Fig. 2F). These genes are targets of atezolizumab and sorafenib, which are approved drugs in the clinical treatment of liver cancer (Fig. 2G) [41]. The overlap between drug targets and spatially co-expressed genes in our database helps to investigate drug sensitivity differences across tissue heterogeneity.

#### Spatial region cluster and cell type comparison

Though newly published ST data often provide spatial region annotations for understanding the anatomical structure of tissues, most previous datasets lack clear annotations. To expand tissue coverage in the spatial2GWAS database, we performed spatial region clustering for 210 unannotated ST slices derived from 10× Visium technology of samples from 23 tissues based on gene expression and spatial distances using BANKSY, with an average of 5 segmented spatial regions per slice. The BANKSY method was reported to segment tissue domains accurately [20]. Although the consistency of clusters and tissue regions still needs validation by experts, the clusters reflect gene expression patterns of specific regions and can provide meaningful spatial features on tissues. To interpret biological differences between clusters, spots in these ST slices were deconvoluted into 13 cell types on average according to results of the stSNV database, and cell type proportions were compared using the Wilcoxon test between GWAS trait-related and unrelated spots across 5784 ST slice-GWAS trait pairs. This analysis identified an average of five differentially represented cell types per pair under a significant threshold of adjusted *P*-values < .05. The cell type proportion of GWAS trait-related and unrelated spots/bins and spatial regions was visualized using box plots and stacked plots to help users directly compare cell composition differences that potentially contribute to the GWAS trait.

For example, we identified six spatial clusters in an ST slice from a human cutaneous squamous cell carcinoma sample, with clusters 0 and 5 associated with a GWAS trait of malignant neoplasm of skin (Supplementary Fig. S3). Spots were deconvoluted into 11 distinct types of cells, such as B cells, T cells, and macrophages. The proportions of B cells, dendritic cells, and endothelial cells were significantly lower in trait-related spots compared to unrelated spots, as well as in the cancer-associated clusters 0 and 5 region compared to unrelated clusters. In contrast, the proportion of fibroblasts was higher in trait-related spots and clusters (Fig. 2H). The specific microenvironment of these cells may contribute to the immune exclusion, invasion, and metastasis of tumors [42–44]. The example shows that clusters on ST slices represent specific spatial features of cell type composition related to GWAS traits and are valuable for interpreting differences across regions contributing to diseases, especially in cancer.

#### Differentially expressed genes and enriched GO terms and KEGG pathways

To further investigate biological dysfunctions associated with GWAS trait-related spatial regions, we identified DEGs between GWAS trait-related and unrelated spots/bins as well as GWAS trait-related and unrelated spatial regions. Analysis at the spot/bin level revealed 15 752 DEGs across 27 217 ST slice-GWAS trait pairs in 1167 slices from 39 tissues. On average, each tissue exhibited 1838 DEGs in 697 ST slice-GWAS trait pairs. At the spatial region level, 16 219 DEGs across 26 670 ST slice-GWAS trait pairs in 1126 slices from 39 tissues were identified. On average, each tissue exhibited 3211 DEGs in 683 ST slice-GWAS trait pairs. For the selected ST slice-GWAS trait pair, tables of DEGs, including fold change values of gene expression and adjusted *P*-values, are accessible on the result page. Based on the DEGs, subsequent enrichment analysis of DEGs identified GO terms and KEGG pathways at both spot/bin and spatial region levels, and the top 15 enriched results were visualized using bubble plots. According to the differentially activated biological processes in GWAS trait-related regions, users can infer potential spatial mechanisms that contribute to complex diseases.

For example, in the above examples of cortical Layer 1 in four human brain ST slices associated with the GWAS trait of AD, we identified AD-related genes such as APP, PSEN1, and APOE differently expressed between Layer 1 and other cortical layers and white matter. Based on the DEGs, pathways such as “AD,” “oxidative phosphorylation,” and “pathways of neurodegeneration” were enriched in all four pairs in the AD-related Layer 1 region (Supplementary Fig. S4, Fig. 2I), which may contribute to the pathogenesis of AD [45, 46]. Additionally, GO terms such as “amyloid-beta formation,” “regulation of neuron projection development,” and “synaptic vesicle cycle” were also enriched in Layer 1 (Supplementary Fig. S5), which participate in AD by critical metabolic and functional dysregulation [47]. These examples indicate that the downstream DEGs and enrichment analyses reveal spatially specific genes and activated biological processes related to high-level phenotypes, facilitating an understanding of disease mechanisms in a spatial manner.

#### Directional cell–cell communication of GWAS trait-associated genes

Compared to single-cell resolution data, ST provides additional information about the spatial distances between spots or cells, facilitating the investigation of cell–cell communication and directional signaling within specific environments with a distance restriction. By combining known ligand–receptor interactions, gene expression data, and spatial distance information from ST slices, the COMMOT method identified 1098 ligand–receptor interactions related to GWAS-associated genes and their co-expressed genes across 26 133 ST slice-GWAS trait pairs. The results cover 38 tissues and 829 slices, with an average of 293 ligand–receptor interactions and 687 ST slice-GWAS trait pairs per tissue. The results are visualized on the result page and can be selected using the ligand–receptor pairs. Integrating with the spatial region distribution in tissues, users can investigate the activation and biochemical signaling direction of ligand–receptor interactions within GWAS trait-associated regions and across related and unrelated regions to spatially explain the impact of signaling on high-level phenotypes.

For example, in the mouse brain ST slice, the chronotype-associated gene BDNF was involved in a ligand–receptor pair of Bdnf-Ntrk2. We identified that spatial regions of Layer 2/3, dentate gyrus, hippocampus, and inhibitory were associated with chronotype. Studies have reported that these regions participate in sleep generation and homeostatic sleep regulation [48–50]. BDNF is a kind of neurotrophin that is involved in the process of activity-dependent plasticity in the brain, plays an important role in neurological disorders, and serves as a drug target [50, 51]. The cell–cell communication of Bdnf-Ntrk2 showed significant signaling interactions between chronotype-related and unrelated spatial regions, such as an outward direction from the hippocampus (Fig. 2J). In sleep-deprived rats, BDNF was found to be a converging point for distinct signaling pathways on hippocampal neurogenesis [52]. The results in the spatial2GWAS pinpoint the directional spatial effect of BDNF as a ligand on chronotype. As shown in the example, the cell–cell communication analysis can supplement spatial direction information for biochemical signaling within and across spatial regions based on ligand–receptor pairs to explain downstream effects on diseases.

### Statistics interface

The “Statistics” page shows statistical information of ST slices and GWAS data using interactive pie charts, bar plots, and box plots, such as the species, tissue, technology, disease, spot number, and GWAS category, giving users a clear understanding of the data available in the spatial2GWAS database. The detailed values, such as counts of bars and quartiles of boxes, are shown when hovering the mouse on the statistic plots.

### Case study

**Figure 3.**
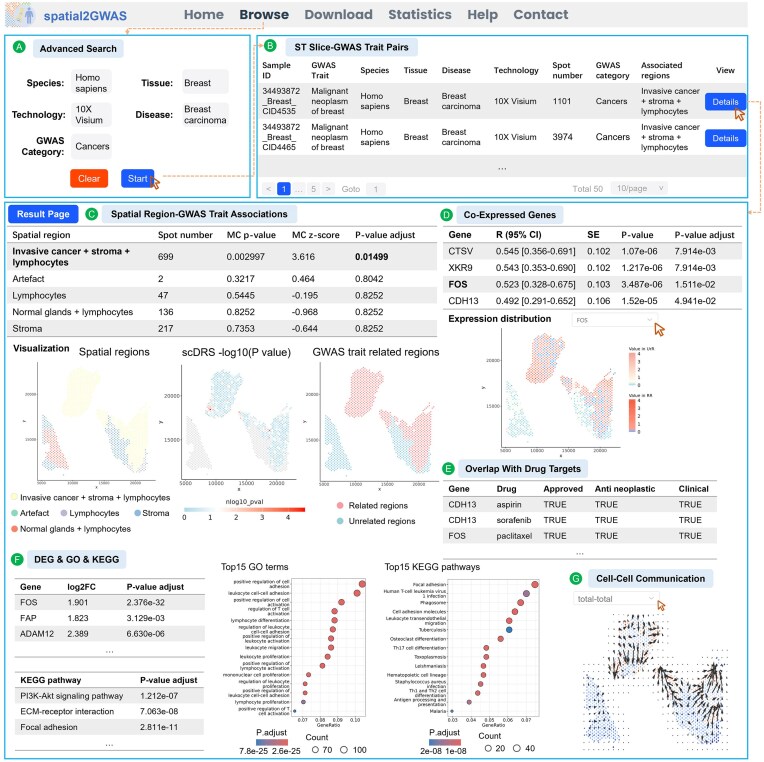
A case study of a breast carcinoma ST slice and GWAS trait “Cancer register—Type of cancer: ICD10: C50 Malignant neoplasm of breast” using spatial2GWAS. (**A**) Utilized the “Advanced search” box on the “Browse” page to search by “Species,” “Tissue,” “Technology,” “Disease,” and “GWAS Trait” categories. (**B**) Search results displaying ST slice-GWAS trait pairs. (**C**) Table of spatial regions and GWAS trait associations, with corresponding plots: annotated spatial regions (left), *P*-values of spots in GWAS trait-related regions calculated by scDRS (middle), and spatial regions related and unrelated to the GWAS trait (right). (**D**) Genes co-expressed with GWAS trait-associated genes and gene expression distribution of FOS. RR: GWAS trait-related regions; UrR: GWAS trait-unrelated regions. (**E**) Overlap between co-expressed genes and drug targets. (**F**) DEGs between trait-related and unrelated regions and the top 15 enriched GO/KEGG processes. (**G**) Directional cell–cell communication signaling in the spatial context, based on total ligand-receptor interactions.

Breast cancer remains a major global health threat for women. Mutations in the genome have been found to account for 5%–16% of breast cancer cases, increasing predisposition risk while affecting drugs’ efficiency and toxicity [53, 54]. In the spatial2GWAS database, we collected gene expression and coordinate data of ST slices from breast cancer samples sequenced using the 10× Visium platform [55]. Pathological annotations were categorized into morphological classes, including normal glands, stroma, lymphocytes, and invasive cancer spots, as defined in the SpatialRef database [15]. Subsequently, associations between spots/spatial regions and GWAS traits related to cancer were analyzed. These results are accessible via the “Advanced search” box on the “Browse” page using the following parameters: “Species: Homo sapiens,” “Tissue: Breast,” “Technology: 10× Visium,” “Disease: Breast carcinoma,” and “GWAS category: Cancers” (Fig. 3A). A total of 50 ST slice-GWAS trait pairs were identified (Fig. 3B).

The ST slice with ID “34493 872_Breast_CID4535” and GWAS trait “Cancer register—Type of cancer: ICD10: C50 Malignant neoplasm of breast” was selected as an example. The ST slice originated from an untreated estrogen receptor positive (ER+) invasive lobular carcinoma patient. The GWAS summary data were derived from the UK Biobank’s European cohort, comprising 10 420 cases and 44 358 controls, with 9 007 627 SNPs tested in these individuals. After preprocessing, the top 1000 breast cancer-associated genes and 1101 spatial spots were retained for further analysis. Detailed results can be accessed by clicking the “Details” button on the table. Using the scDRS workflow, the spatial region annotated as “invasive cancer + stroma + lymphocytes” showed a significant association with the breast cancer GWAS trait (adjusted *P*-value < .05) (Fig. 3C). In the original study, this region was characterized by immunosuppressive signaling, playing a critical role in immune regulation within the tumor microenvironment and offering insights into anti-tumor immune evasion mechanisms that influence clinical outcomes [55].

At the spot level, four genes co-expressed with breast cancer-associated genes were identified (Fig. 3D). Cathepsin V (CTSV), overexpressed in ER+ breast cancer, promotes tumor cell invasion and proliferation and is associated with poor prognosis. Targeting CTSV through histone stabilization could disrupt cell cycle progression and represent a novel therapeutic strategy [56]. Cadherin-13 (CDH13), a tumor suppressor gene, exhibits methylation linked to increased metastatic potential and reduced tamoxifen sensitivity in invasive breast carcinomas [57]. Fos proto-oncogene, AP-1 transcription factor subunit (FOS) suppresses tumor growth in triple-negative breast cancer while enhancing inflammatory responses that recruit neutrophils [58]. XK Related 9 (XKR9), associated with cancer survival outcomes, represents a potential target for cancer immunotherapy [59]. Notably, the co-expressed gene FOS is a target for the approved anti-neoplastic drug paclitaxel, widely used in breast cancer treatment (Fig. 3E) [60].

At the spatial region level, we identified DEGs between GWAS trait-related and unrelated spatial regions and enriched GO terms and KEGG pathways (Fig. 3F). The co-expressed gene FOS was upregulated in the “invasive cancer + stroma + lymphocytes” spatial region, potentially suppressing tumor development and enhancing response to paclitaxel treatment. Enrichment analysis highlighted key pathways, such as “PI3K-Akt signaling pathway,” “ECM-receptor interaction,” and “Focal adhesion,” which are critical for breast tumor progression, metastasis, and prognosis [61–63].

Finally, we visualized directional cell–cell communication in the spatial context. Users can first examine total ligand–receptor interactions by selecting “total–total” to obtain an overview of biochemical signaling in the tumor microenvironment (Fig. 3G). On the breast cancer ST slice, significant signaling activity was observed at the boundary of the “invasive cancer + stroma + lymphocytes” spatial region. Breast cancer-associated pathways such as VEGF, PARs, and COMPLEMENT were activated in the region (Supplementary Fig. S6A–C). For instance, the GWAS trait-associated gene FLT1, involved in the VEGFA-FLT1 ligand–receptor interaction within the VEGF pathway (Supplementary Fig. S6D), has been found to be a therapeutic target for PARP inhibitor resistance [64]. These findings demonstrate that spatial2GWAS can uncover spatial characteristics of breast cancer, aiding in the prediction of disease progression and therapeutic response. As a comprehensive resource, spatial2GWAS bridges ST and GWAS data to advance understanding of tumor pathogenesis across tissue regions, offering translational potential for precision oncology.

## Discussion

The spatial2GWAS database represents a significant advancement in integrating ST with GWAS data, addressing the critical gap between spatial architectures in tissues and genome-wide association findings and offering spatially resolved insights into high-level phenotypes. Through paired analysis of ST slices and GWAS data, we identified 47 492 significant spatial regions, such as white matter, cortical layers 1–6 in the brain, and immune niches (such as germinal center and T cell zone) in the tonsil, specifically associated with GWAS traits related to corresponding tissues, including human complex diseases like mental disorders and cancers. The genes whose expression was correlated with the expression of GWAS trait-associated genes (calculated as disease scores by scDRS) in trait-related spots/bins were defined as co-expressed genes to be the potential markers of the trait in spatial. The overlap between these markers and drug targets indicated that the integration of ST and GWAS data may provide a clue for treatment response and drug resistance from the tissue architecture aspect. To further investigate the spatially activated biological processes that contribute to high-level phenotypes, we enriched GO/KEGG terms based on DEGs between GWAS trait-related and unrelated spatial regions to propose underlying mechanisms, especially for diseases. Using the method that infers cell–cell communication based on ligand–receptor interactions involving GWAS trait-associated and co-expressed genes, we visualized signaling directions within and across spatial regions to reveal the relationship between biochemical interactions and the tissue microenvironment. Thus, spatial2GWAS bridges the resolution gap of GWAS and bulk/single-cell RNA sequencing data, offering a valuable resource for understanding the biological functions of spatial regions associated with GWAS traits.

The spatial2GWAS database contains 1196 ST slices across 39 tissues from human and mouse samples, with 25.0% derived from skin in human samples and 34.2% from spinal cord in mouse samples (Supplementary Fig. S7A). Mouse samples are included in the database for the over 80% correlation in tissue-level expression across genes between mice and humans [8]. The ST data were derived from five widely used technologies (Supplementary Fig. S7B), including low-resolution traditional ST (100 μm per spot) and high-resolution stereo-seq (500 nm each bin), ensuring broad application of workflows for analysis. The ST slices encompass not only healthy tissues but also 49 disease conditions, such as amyotrophic lateral sclerosis (19.9%) and AD (12.0%) (Supplementary Fig. S7C). These ST data are matched with 812 GWAS traits across 18 phenotype categories (e.g. 24.0% in mental disorders and 23.2% in immune diseases) by tissue type to enhance the interpretability of association results between spatial regions and GWAS traits (Supplementary Fig. S7D). On 65.9% of ST slices, only one spatial region was associated with the GWAS trait, indicating a specific spatial characteristic toward phenotype within tissue (Supplementary Fig. S7E). The diverse datasets provide a comprehensive landscape of spatial regions linked to high-level phenotypes, supporting research in disease biology, precision medicine, and environmental genomics.

With the rapid expansion of GWAS trait coverage, increased resolution of ST data, broader diversity of ST-sampled tissue types, and enhanced regional annotations, spatial2GWAS will continuously integrate newly published datasets to analyze associations between a wider range of phenotypes and tissues. The spatial2GWAS workflow, designed to accept standardized ST and GWAS data formats, can be easily applied to new datasets. In the current version of spatial2GWAS, most GWAS data are derived from European populations (767/812). To address population-specific genetic variation, we will collect GWAS data from diverse populations and match the population origins with those of ST samples, enabling population-specific analyses. Additionally, the majority of ST slices are from mouse tissues (792/1196), structural and functional differences limit the relevance results transfer from mouse to human. Future data updates will prioritize human-derived ST data. The data expansion will help to precisely elucidate spatial biological mechanisms underlying phenotypes, particularly complex human diseases.

In the current version of spatial2GWAS, users can browse, search, and visualize associations between spatial regions and GWAS traits, and investigate differences between trait-related and unrelated regions according to downstream analysis results. To improve the usability of the database, the next version will add interactive plots to facilitate exploration of gene expression, biological pathways, and cell–cell communication in spatial regions of interest. We will also implement online tools on the web, accepting user-uploaded data and carrying out data preprocessing, spatial region and GWAS trait association identification, and downstream functional analysis in optional modules to support the establishment of personalized workflows. The spatial2GWAS database, currently applied analysis only to a single ST slice, lacks three-dimensional spatial relationships across ST slices. Further updates will integrate tools for three-dimensional reconstruction [65, 66] to align slices from the same sample and refine three-dimensional spatial blocks and phenotype associations across multi-slice contexts. Additionally, spatial2GWAS provides correlation insights rather than causality between spatial regions and phenotypes. This is a limitation shared by most GWAS-integrated resources, which calls for the development of new methodologies [8, 18]. In summary, the spatial2GWAS database will serve as an important resource for establishing biological hypotheses related to the prediction and prognosis of complex diseases at spatial resolution in the future.

## Supplementary Material

gkaf1047_Supplemental_Files

## Data Availability

Spatial2GWAS is a freely accessible database resource open to all users without registration or logging in at http://www.spatial2gwas.cn. All analysis results and code have been uploaded to https://zenodo.org/records/16880800.

## References

[1] Valihrach L , Zucha D, Abaffy P et al. A practical guide to spatial transcriptomics. Mol Aspects Med. 2024; 97:101276 10.1016/j.mam.2024.101276. 38776574

[2] Maynard KR , Collado-Torres L, Weber LM et al. Transcriptome-scale spatial gene expression in the human dorsolateral prefrontal cortex. Nat Neurosci. 2021; 24:425–36. 10.1038/s41593-020-00787-0. 33558695 PMC8095368

[3] Gulati GS , D’Silva JP, Liu Y et al. Profiling cell identity and tissue architecture with single-cell and spatial transcriptomics. Nat Rev Mol Cell Biol. 2025; 26:11–31. 10.1038/s41580-024-00768-2. 39169166

[4] Tam V , Patel N, Turcotte M et al. Benefits and limitations of genome-wide association studies. Nat Rev Genet. 2019; 20:467–84. 10.1038/s41576-019-0127-1. 31068683

[5] de Leeuw CA , Mooij JM, Heskes T et al. MAGMA: generalized gene-set analysis of GWAS data. PLoS Comput Biol. 2015; 11:e1004219 10.1371/journal.pcbi.1004219. 25885710 PMC4401657

[6] Mishra A , Macgregor S VEGAS2: software for more flexible gene-based testing. Twin Res Hum Genet. 2015; 18:86–91. 10.1017/thg.2014.79. 25518859

[7] Lamparter D , Marbach D, Rueedi R et al. Fast and rigorous computation of gene and pathway scores from SNP-based summary statistics. PLoS Comput Biol. 2016; 12:e1004714 10.1371/journal.pcbi.1004714. 26808494 PMC4726509

[8] Song L , Chen W, Hou J et al. Spatially resolved mapping of cells associated with human complex traits. Nature. 2025; 641:932–41. 10.1038/s41586-025-08757-x. 40108460 PMC12095064

[9] Cao J , Li C, Cui Z et al. Spatial transcriptomics: a powerful tool in disease understanding and drug discovery. Theranostics. 2024; 14:2946–68. 10.7150/thno.95908. 38773973 PMC11103497

[10] Wang S , Greenbaum J, Qiu C et al. Gene interactions analysis of brain spatial transcriptome for Alzheimer’s disease. Genes Dis. 2024; 11:101337 10.1016/j.gendis.2024.101337. 39281834 PMC11402150

[11] Tang S , Liu S, Buchman AS et al. Integrating spatial transcriptomics and snRNA-seq data enhances differential gene expression analysis results of AD-related phenotypes. HGG Adv. 2025; 6:100447. 40329537 10.1016/j.xhgg.2025.100447 PMC12159441

[12] Zhang C , Chen R, Zhang Y Accurate inference of genome-wide spatial expression with iSpatial. Sci Adv. 2022; 8:eabq0990 10.1126/sciadv.abq0990. 36026447 PMC9417177

[13] Miyoshi E , Morabito S, Henningfield CM et al. Spatial and single-nucleus transcriptomic analysis of genetic and sporadic forms of Alzheimer’s disease. Nat Genet. 2024; 56:2704–17. 10.1038/s41588-024-01961-x. 39578645 PMC11631771

[14] Fan Z , Luo Y, Lu H et al. SPASCER: spatial transcriptomics annotation at single-cell resolution. Nucleic Acids Res. 2023; 51:D1138–49. 10.1093/nar/gkac889. 36243975 PMC9825565

[15] Cui T , Li Y-Y, Li B-L et al. SpatialRef: a reference of spatial omics with known spot annotation. Nucleic Acids Res. 2025; 53:D1215–23. 10.1093/nar/gkae892. 39417483 PMC11701618

[16] Yang C , Liu Y, Wang X et al. stSNV: a comprehensive resource of SNVs in spatial transcriptome. Nucleic Acids Res. 2025; 53:D1224–34. 10.1093/nar/gkae945. 39470702 PMC11701523

[17] Li F , Song X, Fan W et al. SPathDB: a comprehensive database of spatial pathway activity atlas. Nucleic Acids Res. 2025; 53:D1205–14. 10.1093/nar/gkae1041. 39546631 PMC11701687

[18] Yin M , Feng C, Yu Z et al. sc2GWAS: a comprehensive platform linking single cell and GWAS traits of human. Nucleic Acids Res. 2025; 53:D1151–61. 10.1093/nar/gkae1008. 39565208 PMC11701642

[19] Xu Z , Wang W, Yang T et al. STOmicsDB: a comprehensive database for spatial transcriptomics data sharing, analysis and visualization. Nucleic Acids Res. 2024; 52:D1053–61. 10.1093/nar/gkad933. 37953328 PMC10767841

[20] Singhal V , Chou N, Lee J et al. BANKSY unifies cell typing and tissue domain segmentation for scalable spatial omics data analysis. Nat Genet. 2024; 56:431–41. 10.1038/s41588-024-01664-3. 38413725 PMC10937399

[21] Wolf FA , Angerer P, Theis FJ SCANPY: large-scale single-cell gene expression data analysis. Genome Biol. 2018; 19:15 10.1186/s13059-017-1382-0. 29409532 PMC5802054

[22] Zheng X , Tian Z, Che X et al. DMRdb: a disease-centric mendelian randomization database for systematically assessing causal relationships of diseases with genes, proteins, CpG sites, metabolites and other diseases. Nucleic Acids Res. 2025; 53:D1363–71. 10.1093/nar/gkae853. 39351893 PMC11701675

[23] Sollis E , Mosaku A, Abid A et al. The NHGRI-EBI GWAS catalog: knowledgebase and deposition resource. Nucleic Acids Res. 2023; 51:D977–85. 10.1093/nar/gkac1010. 36350656 PMC9825413

[24] Kurki MI , Karjalainen J, Palta P et al. FinnGen provides genetic insights from a well-phenotyped isolated population. Nature. 2023; 613:508–18. 10.1038/s41586-022-05473-8. 36653562 PMC9849126

[25] Kent WJ , Sugnet CW, Furey TS et al. The human genome browser at UCSC. Genome Res. 2002; 12:996–1006. 10.1101/gr.229102. 12045153 PMC186604

[26] Zhang MJ , Hou K, Dey KK et al. Polygenic enrichment distinguishes disease associations of individual cells in single-cell RNA-seq data. Nat Genet. 2022; 54:1572–80. 10.1038/s41588-022-01167-z. 36050550 PMC9891382

[27] Cannon M , Stevenson J, Stahl K et al. DGIdb 5.0: rebuilding the drug-gene interaction database for precision medicine and drug discovery platforms. Nucleic Acids Res. 2024; 52:D1227–35. 10.1093/nar/gkad1040. 37953380 PMC10767982

[28] Knox C , Wilson M, Klinger CM et al. DrugBank 6.0: the DrugBank knowledgebase for 2024. Nucleic Acids Res. 2024; 52:D1265–75. 10.1093/nar/gkad976. 37953279 PMC10767804

[29] Stuart T , Butler A, Hoffman P et al. Comprehensive integration of single-cell data. Cell. 2019; 177:1888–1902. 10.1016/j.cell.2019.05.031. 31178118 PMC6687398

[30] Ashburner M , Ball CA, Blake JA et al. Gene Ontology: tool for the unification of biology. The Gene Ontology consortium. Nat Genet. 2000; 25:25–9. 10.1038/75556. 10802651 PMC3037419

[31] Ontology Consortium G Aleksander SA , Balhoff J et al. The Gene Ontology knowledgebase in 2023. Genetics. 2023; 224:iyad031 10.1093/genetics/iyad031. 36866529 PMC10158837

[32] Kanehisa M , Furumichi M, Sato Y et al. KEGG: biological systems database as a model of the real world. Nucleic Acids Res. 2025; 53:D672–7. 10.1093/nar/gkae909. 39417505 PMC11701520

[33] Wu T , Hu E, Xu S et al. clusterProfiler 4.0: a universal enrichment tool for interpreting omics data. Innovation (Camb). 2021; 2:100141. 34557778 10.1016/j.xinn.2021.100141 PMC8454663

[34] Cang Z , Zhao Y, Almet AA et al. Screening cell–cell communication in spatial transcriptomics via collective optimal transport. Nat Methods. 2023; 20:218–28. 10.1038/s41592-022-01728-4. 36690742 PMC9911355

[35] Jin S , Guerrero-Juarez CF, Zhang L et al. Inference and analysis of cell–cell communication using CellChat. Nat Commun. 2021; 12:1088 10.1038/s41467-021-21246-9. 33597522 PMC7889871

[36] Cohen-Kashi Malina K , Tsivourakis E, Kushinsky D et al. NDNF interneurons in layer 1 gain-modulate whole cortical columns according to an animal’s behavioral state. Neuron. 2021; 109:2150–64. 10.1016/j.neuron.2021.05.001. 34038743

[37] Ibrahim LA , Huang S, Fernandez-Otero M et al. Bottom-up inputs are required for establishment of top-down connectivity onto cortical layer 1 neurogliaform cells. Neuron. 2021; 109:3473–85. 10.1016/j.neuron.2021.08.004. 34478630 PMC9316418

[38] Gazestani V , Kamath T, Nadaf NM et al. Early Alzheimer’s disease pathology in human cortex involves transient cell states. Cell. 2023; 186:4438–53. 10.1016/j.cell.2023.08.005. 37774681 PMC11107481

[39] Liongue C , Ratnayake T, Basheer F et al. Janus kinase 3 (JAK3): a critical conserved node in immunity disrupted in immune cell cancer and immunodeficiency. Int J Mol Sci. 2024; 25:2977 10.3390/ijms25052977. 38474223 PMC10932405

[40] Qiu X , Zhou T, Li S et al. Spatial single-cell protein landscape reveals vimentin^high^ macrophages as immune-suppressive in the microenvironment of hepatocellular carcinoma. Nat Cancer. 2024; 5:1557–78. 10.1038/s43018-024-00824-y. 39327501

[41] Llovet JM , Castet F, Heikenwalder M et al. Immunotherapies for hepatocellular carcinoma. Nat Rev Clin Oncol. 2022; 19:151–72. 10.1038/s41571-021-00573-2. 34764464

[42] Bindea G , Mlecnik B, Tosolini M et al. Spatiotemporal dynamics of intratumoral immune cells reveal the immune landscape in human cancer. Immunity. 2013; 39:782–95. 10.1016/j.immuni.2013.10.003. 24138885

[43] Biffi G , Tuveson DA Diversity and biology of cancer-associated fibroblasts. Physiol Rev. 2021; 101:147–76. 10.1152/physrev.00048.2019. 32466724 PMC7864232

[44] Yang L , Joseph S, Sun T et al. TAK1 regulates endothelial cell necroptosis and tumor metastasis. Cell Death Differ. 2019; 26:1987–97. 10.1038/s41418-018-0271-8. 30683914 PMC6748133

[45] Cunnane SC , Trushina E, Morland C et al. Brain energy rescue: an emerging therapeutic concept for neurodegenerative disorders of ageing. Nat Rev Drug Discov. 2020; 19:609–33. 10.1038/s41573-020-0072-x. 32709961 PMC7948516

[46] Parhizkar S , Holtzman DM APOE mediated neuroinflammation and neurodegeneration in Alzheimer’s disease. Semin Immunol. 2022; 59:101594 10.1016/j.smim.2022.101594. 35232622 PMC9411266

[47] Ovsepian SV , O’Leary VB, Zaborszky L et al. Synaptic vesicle cycle and amyloid β: biting the hand that feeds. Alzheimers Dement. 2018; 14:502–13. 10.1016/j.jalz.2018.01.011. 29494806

[48] Dong Y , Li J, Zhou M et al. Cortical regulation of two-stage rapid eye movement sleep. Nat Neurosci. 2022; 25:1675–82. 10.1038/s41593-022-01195-2. 36396977

[49] Boyce R , Glasgow SD, Williams S et al. Causal evidence for the role of REM sleep theta rhythm in contextual memory consolidation. Science. 2016; 352:812–6. 10.1126/science.aad5252. 27174984

[50] Amanollahi M , Jameie M, Heidari A et al. The dialogue between neuroinflammation and adult neurogenesis: mechanisms involved and alterations in neurological diseases. Mol Neurobiol. 2023; 60:923–59. 10.1007/s12035-022-03102-z. 36383328

[51] Saitoh K , Furihata R, Kaneko Y et al. Association of serum BDNF levels and the BDNF Val66Met polymorphism with the sleep pattern in healthy young adults. PLoS One. 2018; 13:e0199765 10.1371/journal.pone.0199765. 29944703 PMC6019675

[52] Rossi C , Angelucci A, Costantin L et al. Brain-derived neurotrophic factor (BDNF) is required for the enhancement of hippocampal neurogenesis following environmental enrichment. Eur J of Neurosci. 2006; 24:1850–6. 10.1111/j.1460-9568.2006.05059.x. 17040481

[53] Shiovitz S , Korde LA Genetics of breast cancer: a topic in evolution. Ann Oncol. 2015; 26:1291–9. 10.1093/annonc/mdv022. 25605744 PMC4478970

[54] Moustafa HAM , El-Dakroury WA, Ashraf A et al. SNP’s use as a potential chemotoxicity stratification tool in breast cancer: from bench to clinic. Funct Integr Genomics. 2025; 25:93 10.1007/s10142-025-01602-4. 40261508

[55] Wu SZ , Al-Eryani G, Roden DL et al. A single-cell and spatially resolved atlas of human breast cancers. Nat Genet. 2021; 53:1334–47. 10.1038/s41588-021-00911-1. 34493872 PMC9044823

[56] Sereesongsaeng N , Burrows JF, Scott CJ et al. Cathepsin V regulates cell cycle progression and histone stability in the nucleus of breast cancer cells. Front Pharmacol. 2023; 14:1271435 10.3389/fphar.2023.1271435. 38026973 PMC10657903

[57] Xu J , Shetty PB, Feng W et al. Methylation of HIN-1, RASSF1A, RIL and CDH13 in breast cancer is associated with clinical characteristics, but only RASSF1A methylation is associated with outcome. BMC Cancer. 2012; 12:243 10.1186/1471-2407-12-243. 22695491 PMC3476972

[58] Yan S , Zhao W, Du J et al. C-FOS promotes the formation of neutrophil extracellular traps and the recruitment of neutrophils in lung metastasis of triple-negative breast cancer. J Exp Clin Cancer Res. 2025; 44:108 10.1186/s13046-025-03370-2. 40148973 PMC11951605

[59] Li Y , Pang X, Cui Z et al. Genetic factors associated with cancer racial disparity—an integrative study across twenty-one cancer types. Molecular Oncology. 2020; 14:2775–86. 10.1002/1878-0261.12799. 32920960 PMC7607166

[60] Roskoski R Targeted and cytotoxic inhibitors used in the treatment of breast cancer. Pharmacol Res. 2024; 210:107534 10.1016/j.phrs.2024.107534. 39631485

[61] Zhu K , Wu Y, He P et al. PI3K/AKT/mTOR-targeted therapy for breast cancer. Cells. 2022; 11:2508 10.3390/cells11162508. 36010585 PMC9406657

[62] Bao Y , Wang L, Shi L et al. Transcriptome profiling revealed multiple genes and ECM-receptor interaction pathways that may be associated with breast cancer. Cell Mol Biol Lett. 2019; 24:38 10.1186/s11658-019-0162-0. 31182966 PMC6554968

[63] Luo M , Guan J-L Focal adhesion kinase: a prominent determinant in breast cancer initiation, progression and metastasis. Cancer Lett. 2010; 289:127–39. 10.1016/j.canlet.2009.07.005. 19643531 PMC2854647

[64] Weigand M , Hantel P, Kreienberg R et al. Autocrine vascular endothelial growth factor signalling in breast cancer. Evidence from cell lines and primary breast cancer cultures *in vitro*. Angiogenesis. 2005; 8:197–204. 10.1007/s10456-005-9010-0. 16328160

[65] Schott M , León-Periñán D, Splendiani E et al. Open-ST: high-resolution spatial transcriptomics in 3D. Cell. 2024; 187:3953–72. 10.1016/j.cell.2024.05.055. 38917789

[66] Xiao Z , Cui L, Yuan Y et al. 3D reconstruction of a gastrulating human embryo. Cell. 2024; 187:2855–74. 10.1016/j.cell.2024.03.041. 38657603

