# Supplementary material for: Spatial2GWAS: a database for linking spatial transcriptomic regions with GWAS traits
Source: Nucleic Acids Res. 2025 Oct 22;54(D1):D1309–20. doi: 10.1093/nar/gkaf1047 (PMC12807712; doi:10.1093/nar/gkaf1047)
Supplement: gkaf1047_Supplemental_Files [file gkaf1047_supplemental_files.zip › NAR-spatial2GWAS supp Figures - 8.11.docx]

**Supplementary Figures**


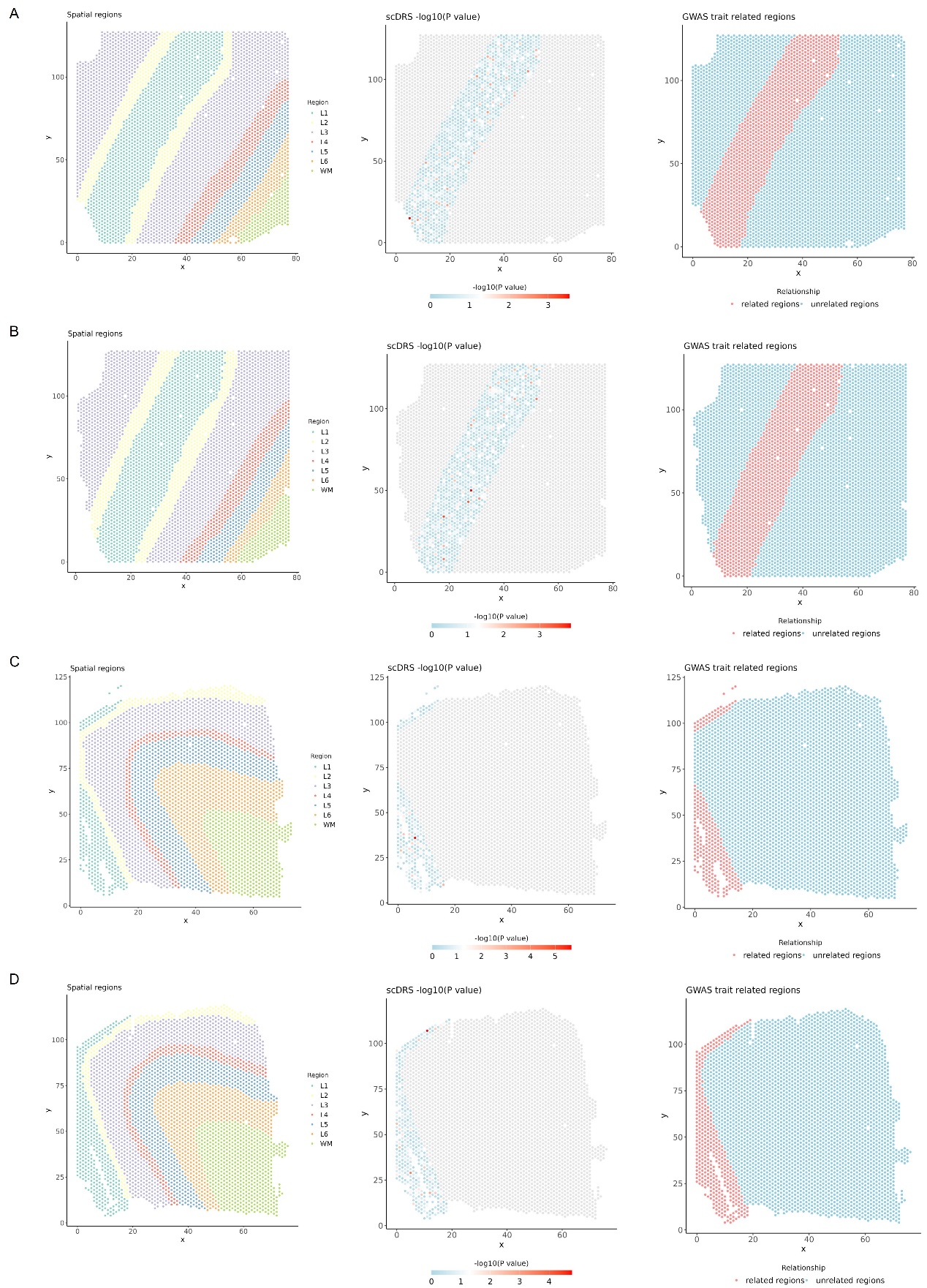


Figure S1. Associations of spatial regions on four spatial transcriptomic slices from human brain samples and the GWAS trait of Alzheimer’s disease. Left: annotated spatial regions; middle: *P*-values of spots calculated by scDRS; right: spatial regions related (L1, spots in red) and unrelated (spots in blue) to Alzheimer’s disease. GWAS ID: SC_G_100. Sample IDs from A to D: 151509, 151510, 151673, 151674.


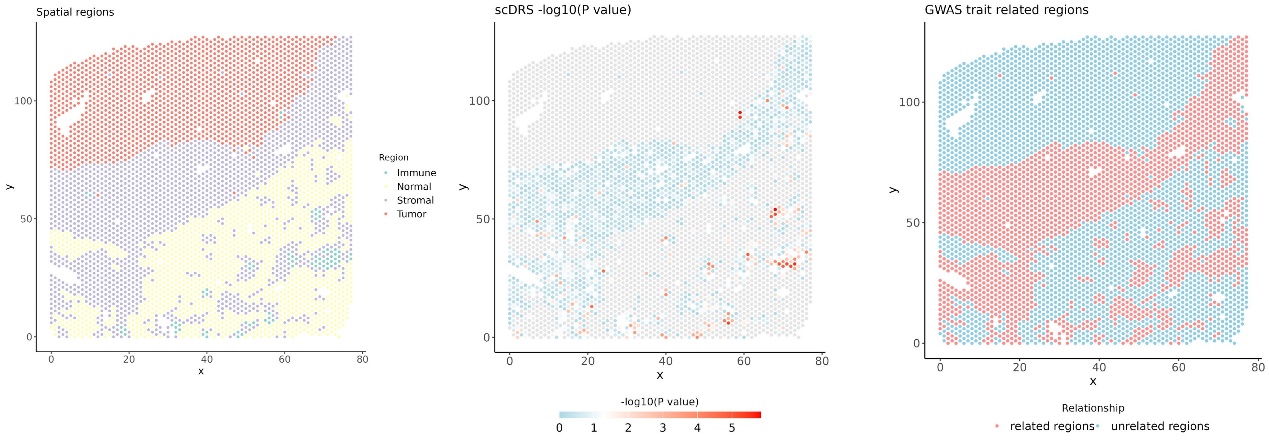


Figure S2. Associations of spatial regions on a spatial transcriptomic slice from a human liver cancer sample and the GWAS trait ”Cancer (diagnosed by doctor)”. Left: annotated spatial regions; middle: *P*-values of spots calculated by scDRS; right: spatial regions related (Immune and Stromal, spots in red) and unrelated (spots in blue) to the GWAS trait. GWAS ID: SC_G_051. Sample ID: HCC-3L.


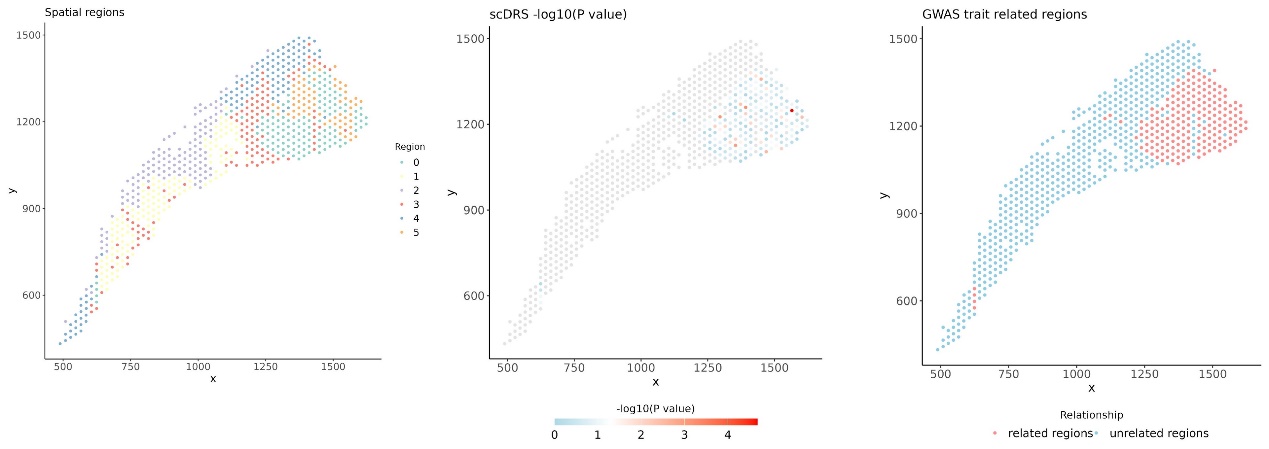


Figure S3. Associations of spatial regions on a spatial transcriptomic slice from a human cutaneous squamous cell carcinoma sample and the GWAS trait of “Diagnoses - main ICD10: C44 Other and unspecified malignant neoplasm of skin”. Left: clusters of spatial regions by BANKSY; middle: *P*-values of spots calculated by scDRS; right: spatial regions related (cluster 0 and 5, spots in red) and unrelated (spots in blue) to malignant neoplasm of skin. GWAS ID: SC_G_015. Sample ID: GSM4565823.


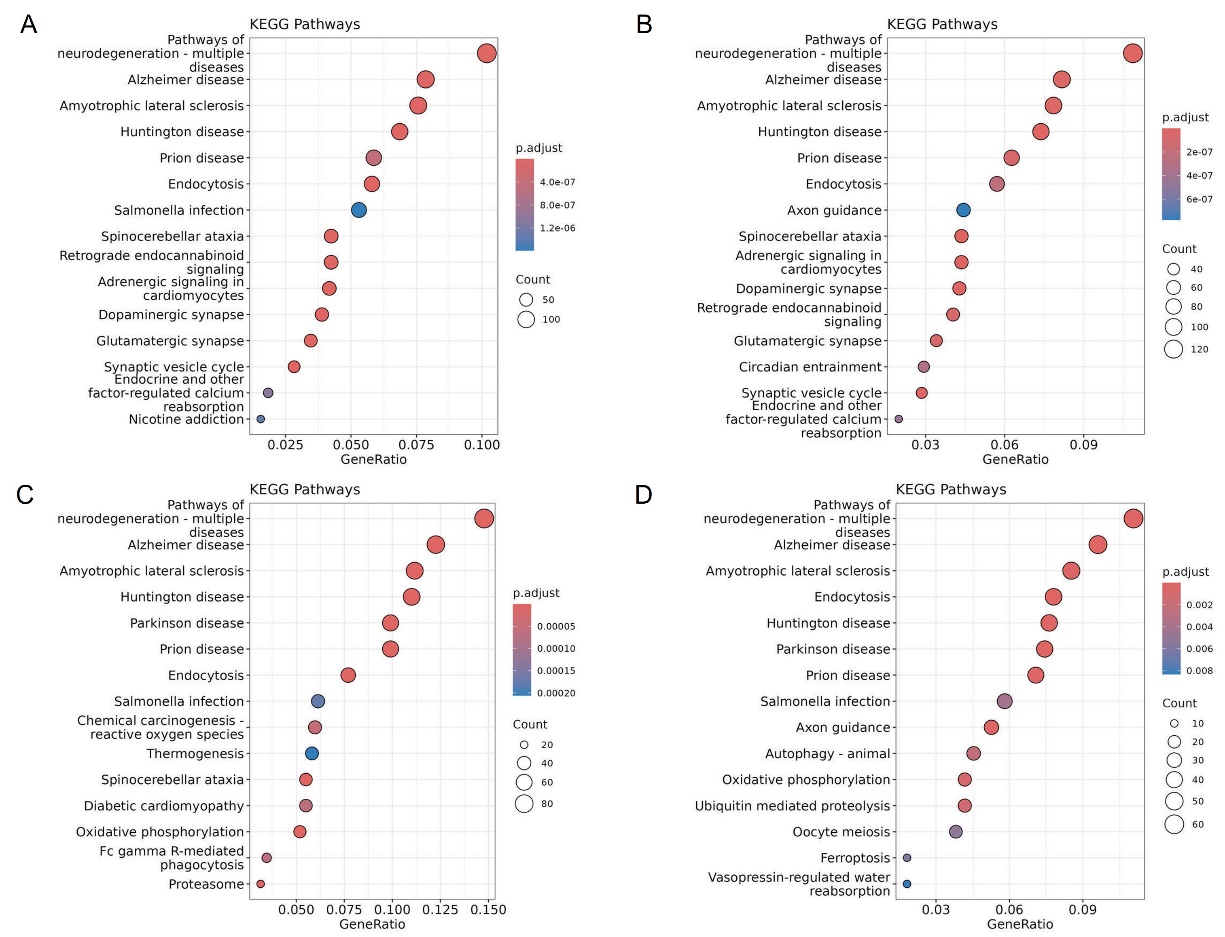


Figure S4. Top 15 enriched KEGG pathways based on differentially expressed genes in Alzheimer’s disease-associated region Layer 1 compared to other cortical layers and white matter. GWAS ID: SC_G_100. Sample IDs from A to D: 151509, 151510, 151673, 151674.


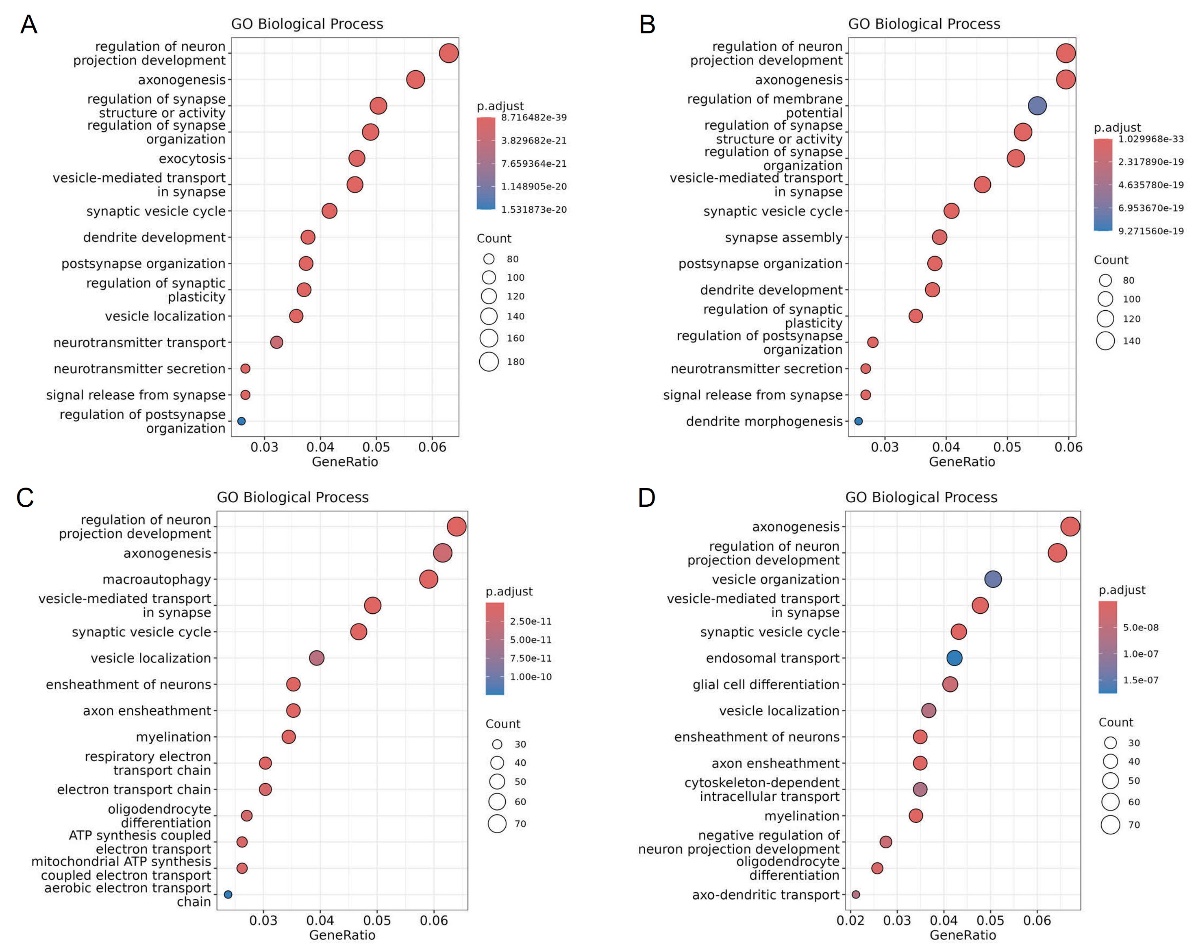


Figure S5. Top 15 enriched GO terms based on differentially expressed genes in Alzheimer’s disease-associated region Layer 1 compared to other cortical layers and white matter. GWAS ID: SC_G_100. Sample IDs from A to D: 151509, 151510, 151673, 151674.


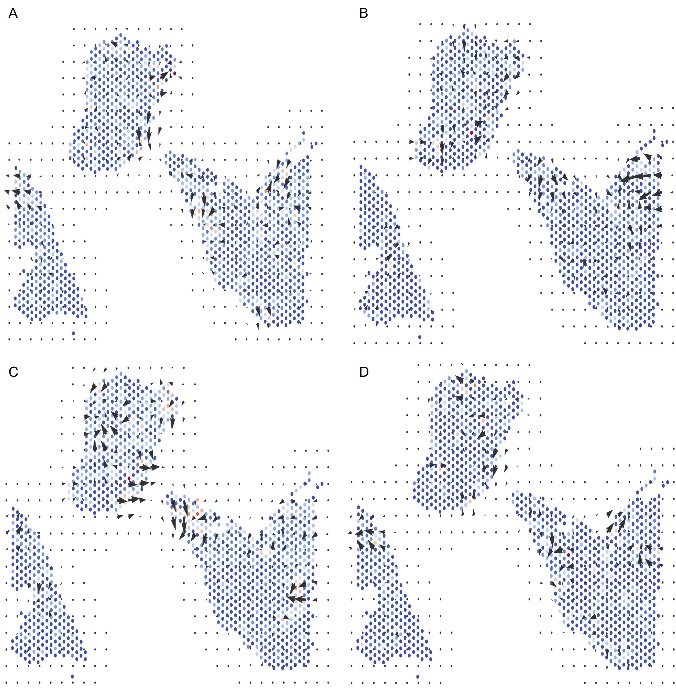


Figure S6. Cell-cell communication visualization based on (A) VEGF, (B) PARs, and (C) COMPLEMENT pathways, and (D) VEGFA-FLT1 ligand-receptor pair in a human breast cancer spatial transcriptomic slice. Sample ID: 34493872_Breast_CID4535.


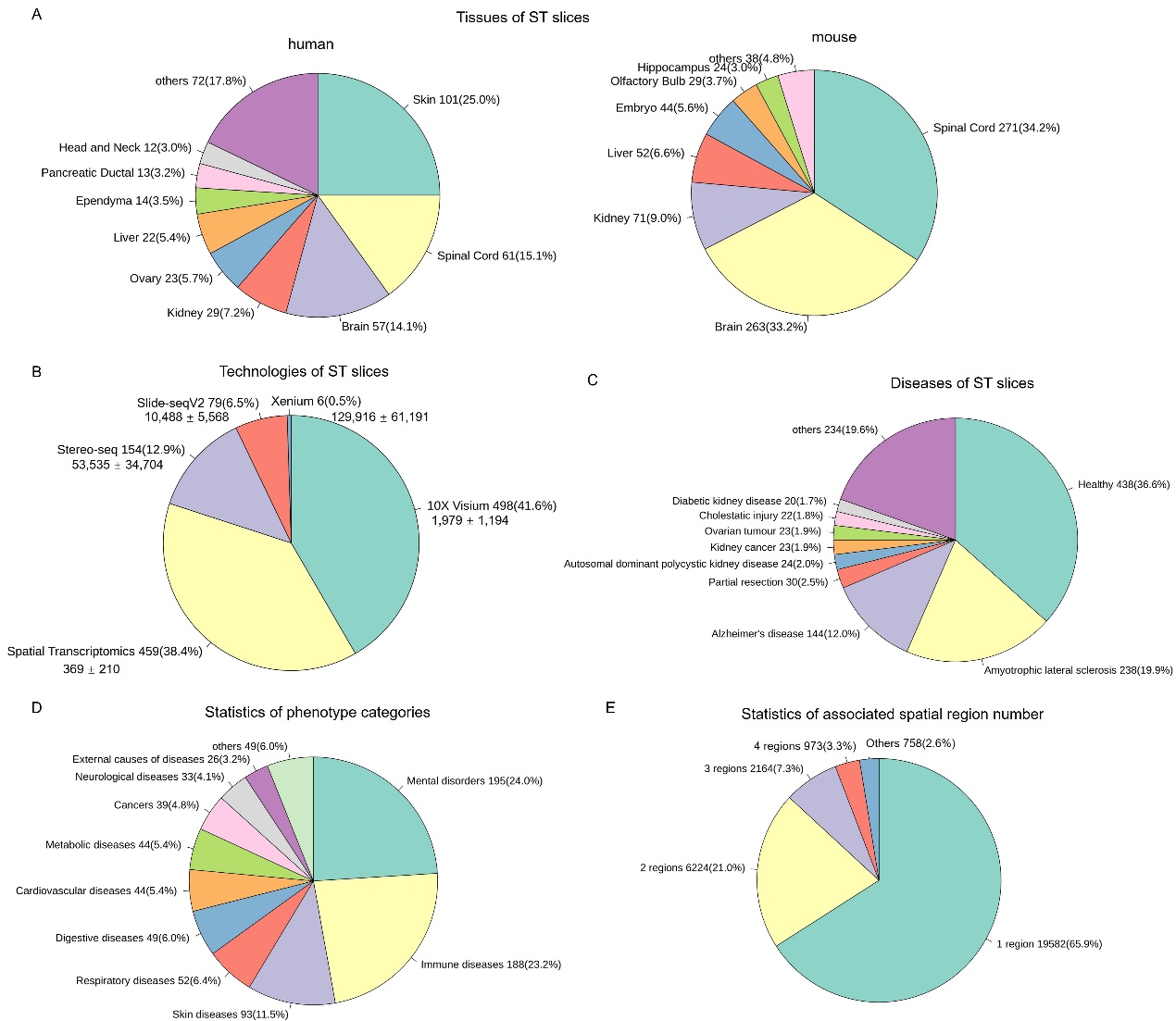


Figure S7. Statistics of data in spatial2GWAS. (A) Proportion of spatial transcriptomic (ST) slices in human tissues (left, top 9 and others) and mouse tissues (right, top 7 and others). (B) Proportion of ST slices derived from five ST technologies, along with the average number of spots/bins per ST slice for each technology (mean ± SD). (C) Proportion of ST slices in disease states (top 9 and others). (D) Proportion of phenotype categories in GWAS summary data (top 9 and others). (E) Proportion of GWAS trait-related spatial regions in ST slice- GWAS trait pairs (number of associated regions from 1 to 4 and others).
